# A neuromorphic system for video object recognition

**DOI:** 10.3389/fncom.2014.00147

**Published:** 2014-11-28

**Authors:** Deepak Khosla, Yang Chen, Kyungnam Kim

**Affiliations:** HRL Laboratories, LLCMalibu, CA, USA

**Keywords:** object detection, object classification, airborne, video image processing, neuromorphic, bio-inspired, low-power, real-time processing

## Abstract

Automated video object recognition is a topic of emerging importance in both defense and civilian applications. This work describes an accurate and low-power neuromorphic architecture and system for real-time automated video object recognition. Our system, Neuormorphic Visual Understanding of Scenes (NEOVUS), is inspired by computational neuroscience models of feed-forward object detection and classification pipelines for processing visual data. The NEOVUS architecture is inspired by the ventral (*what*) and dorsal (*where*) streams of the mammalian visual pathway and integrates retinal processing, object detection based on form and motion modeling, and object classification based on convolutional neural networks. The object recognition performance and energy use of the NEOVUS was evaluated by the Defense Advanced Research Projects Agency (DARPA) under the Neovision2 program using three urban area video datasets collected from a mix of stationary and moving platforms. These datasets are challenging and include a large number of objects of different types in cluttered scenes, with varying illumination and occlusion conditions. In a systematic evaluation of five different teams by DARPA on these datasets, the NEOVUS demonstrated the best performance with high object recognition accuracy and the lowest energy consumption. Its energy use was three orders of magnitude lower than two independent state of the art baseline computer vision systems. The dynamic power requirement for the complete system mapped to commercial off-the-shelf (COTS) hardware that includes a 5.6 Megapixel color camera processed by object detection and classification algorithms at 30 frames per second was measured at 21.7 Watts (W), for an effective energy consumption of 5.45 nanoJoules (nJ) per bit of incoming video. These unprecedented results show that the NEOVUS has the potential to revolutionize automated video object recognition toward enabling practical low-power and mobile video processing applications.

## Introduction

Unmanned platforms are becoming one of the major sources of data for intelligence and surveillance both on and off the battlefield. High resolution and wide field-of-view sensors are resulting in large volume of images and videos that then need to be processed and analyzed. Two problems arise from these emerging trends: (1) High bandwidth is required to send data from the platform to ground stations even with good compression and (2) High workload is imposed on analysts and end-users to process the data. One solution to these problems is to perform on-board automated image and video analysis (e.g., detect, recognize and track objects of interest) to enable better and timely situational awareness, reduce the amount of data to be streamed, and thus reduce the end-user workload. However, rapid and accurate situational awareness is virtually impossible on-board size- and power-constrained mobile platforms using current state of the art video-processing methods. Video data from these platforms typically includes a large number of objects with few pixels on target and occur under changing illumination, occlusion and clutter conditions. Conventional computer vision methods are generally engineered for specific and limited domains, lack robustness and are computationally expensive, making them unsuitable for onboard processing.

This work presents a real-time video object recognition system that is suitable for onboard processing, for example, on unmanned intelligence, surveillance and reconnaissance (ISR) platforms. This work was partially performed under the DARPA Neovision2 program (DARPA, 2011) whose goal was to enable an unattended visual object-recognition capability on unmanned reconnaissance platforms. Our system NEOVUS departs from traditional, domain-specific engineered video-processing capabilities and is instead inspired by neuromorphic algorithms that model visual information processing (Mishkin et al., 1983; Huang and Grossberg, 2010; LeCun et al., 2010). The goal of NEOVUS is to detect and classify objects of interest (e.g., car, truck, person and boat) in videos in real-time, while consuming significantly lower power than state of the art computer vision.

The NEOVUS combines two key capabilities: (1) Object detection that finds potential objects in the image and outlines a bounding box around them. It combines form-based detection using attention approaches to detect entities based on form (e.g., shape, color, intensity) and motion-based detection mediated by spatial attention. (2) Object classification that then classifies the detected objects based on a feed-forward multi-layered convolutional neural network approach (ConvNet) (LeCun et al., 2010; Farabet et al., 2011). The combination of detection followed by classification provides object recognition results. The NEOVUS is implemented in COTS hardware to achieve real-time performance at low size, weight, and power (SWaP). Several components and capabilities of NEOVUS have been previously described by us Chen et al. (2011), Khosla et al. (2013a), Khosla et al. (2013b), Honda et al. (2013). This paper describes the complete system end-to-end, provides additional details of components and key capabilities, and describes in detail the results of DARPA evaluation on urban datasets.

The rest of the paper is organized as follows. Section Architecture describes the NEOVUS architecture. Section Algorithms describes the underlying algorithms for object detection and classification. Section Hardware Mapping describes mapping of the NEOVUS to COTS hardware. Section Results and Discussion describes object recognition performance and energy consumption results from evaluation of the NEOVUS. Finally, Section Conclusions provides conclusions and discusses some future directions of our work.

## Architecture

**Figure 1:**
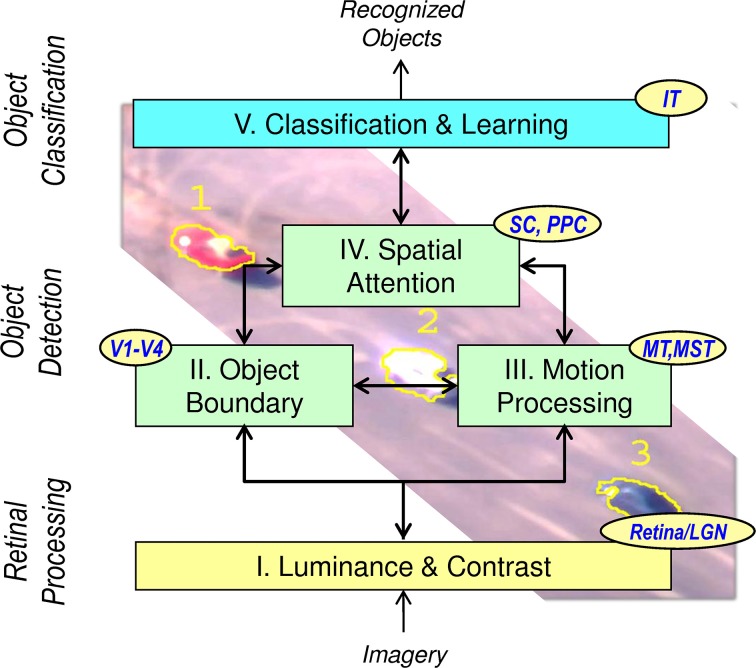
**NEOVUS is an integrated, visual cognition architecture that emulates mammalian dorsal and ventral visual functions**. Five functional components I–V unify the state of the art in retinal processing, object detection and classification. (*LGN, Lateral Geniculate Nucleus; V1-V4, Visual Cortex areas; MT, Middle Temporal; MST, Medial Superior Temporal; SC, Superior Colliculus; PPC, Posterior Parietal Cortex; IT, Infero-Temporal Cortex*).

The NEOVUS is a neuromorphic object-recognition architecture and system that is inspired by the ventral (*what*) and dorsal (*where*) streams of the mammalian visual pathway (Mishkin et al., 1983). It is based on and consistent with neuroscience theories and models of mammalian pathways implicated in visual processing (Mishkin et al., 1983; Huang and Grossberg, 2010). The NEOVUS consists of three primary functions: retinal processing, object detection and object classification, and five components (I–V). Figure 1 shows the architecture that combines retinal processing (I), object detection (II–IV) and object classification (V) for fast and accurate object recognition. Table 1 below summarizes these functional components (I–V).

**Table 1:** **Summary of the five functional components (I–V) in the NEOVUS architecture, their hypothesized brain structures, and functions**.

**Functional component**	**Brain structures**	**Function description**
**I**. Light adaptation (Luminance and Contrast)	Retina-LGN	Light adaptation and contrast enhancement (Werblin and Dowling, 2010). This component was developed under a separate retinal camera hardware effort and is not described in this paper
**II**. Boundary processing	(P-retinal cells)—(LGN Parvo)-(V1 Interblob)-(V2 Interstripe)-V4; (M-retinal cells)—(LGN Parvo)-(V1 Blob)-(V2 Thin Stripe)-V4	Perceptual boundaries and surfaces (lightness and color) are hypothesized to form; complementary edge and surface processes define boundaries across noise occlusions, fill-in featural properties and aid in figure-ground separation (Mishkin et al., 1983; Elder and Zucker, 1998; Huang and Grossberg, 2010)
**III**. Motion processing	Toward posterior parietal cortex via V1, MT, and MST with feedback to V4	Computing object direction and speed further aids figure-ground separation and provides ability to detect objects even under occlusion (Sigala et al., 2005)
**IV**. Spatial attention	SC, LIP, and PPC	Core of the *where* stream; hypothesized formation of attention shrouds and final fused object detections (Mishkin et al., 1983; Huang and Grossberg, 2010)
**V**. Classification and learning	ITa, ITp, and PFC	Core of the *what* stream; hypothesized learning and recognizing object classes (Mishkin et al., 1983; Tanaka, 2000; Ranganath, 2006)

Data flow description and interaction between the components have been previously described (Khosla et al., 2013a). Unlike traditional computer vision approaches (e.g., Felzenszwalb et al., 2010; Kembhavi et al., 2011) that use engineered features and raster-scan processing, this architecture first detects objects and then classifies them based on a set of learned features. The object recognition results are then displayed to the end user for operation and decision-making. In an automated processing application, such results can be used to determine what data are to be transmitted to the end-user, fulfilling the requirements of bandwidth and workload reduction as outlined in the Introduction section. For example, one may wish to transmit sample images of only certain classes of objects (e.g., red car) when the system achieves certain confidence level in its classification results.

## Algorithms

### Object detection

The purpose of object detection is to locate potential object regions in the video and pass them on to the object classification stage. This module detects motion and form by modeling interacting dorsal and ventral pathways based on the two stream models proposed by neuroscientists (Mishkin et al., 1983; Huang and Grossberg, 2010). It needs to operate with a high probability of detection even at reasonably high probability of false positives so as not to miss potential true objects. The false positives are then discarded during the classification stage as background or irrelevant objects. We now describe details of the object detection algorithm.

#### Static object detection

**Figure 2:**
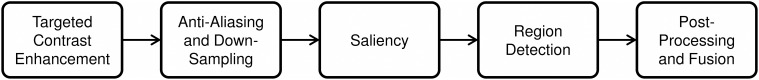
**Workflow of static target detection using form-based attention**.

Static object detection emulates the attentional pathways that detect objects based on their form saliency with respect to the background. The algorithm is motivated by research on using spectral analysis for visual saliency (Hou and Zhang, 2007). Figure 2 shows the block diagram of the major steps in our approach to detecting static objects using the RS method (Honda et al., 2013).

**Figure 3:**
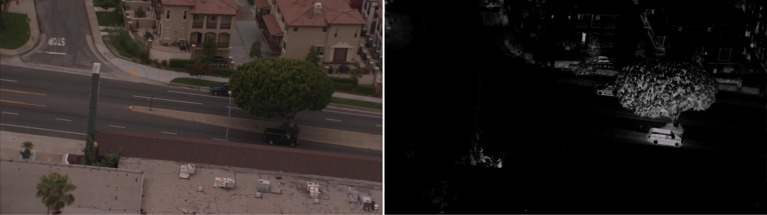
**An illustration of targeted contrast enhancement for color black ([0, 0, 0]) and β = 55**. The low contrast vehicle in the shade of the tree is enhanced so that it clearly stands out from the shadows.

***Targeted contrast enhancement (TCE)***. The original RS approach works only on gray-scale images. This works well on bright objects, but not on dark objects. TCE enhances gray-scale images for specified colors (e.g., black) which then enables us to easily detect objects with these colors. This idea can be extended to any arbitrary color for a potential object of interest. TCE is performed by using an un-normalized Gaussian function with mean value of the target color and variance incorporated into a single user-specified sensitivity parameter β. The color response for pixel value *V*(*c*) and a select set of *M* target colors *T* = {*T_i_*(*c*): *i* ∈ … *M*} is:

$$R(V,T_{i})=\exp\left(-\beta_{i}\sum_{c}{\left(V(c)-T_{i}(c)\right)}^{2}\right),$$

where *c* indexes the color channel and β is a sensitivity parameter for the similarity between that color channel and the target color. Usually β is set to 1.0, but can be a varying value for different colors depending on the application. The response *R* is 1 when the image matches the target color and falls off based on deviation from the target color values. Thus, TCE results in multiple gray-scale response maps, one for each target color. Figure 3 illustrates the effect of TCE for a dark vehicle against dark background.

***Anti-aliasing and down-sampling***. In order to efficiently deal with large images, we down sample the response maps from previous step. Down-sampling can significantly reduce the computational load for subsequent processing. In addition, humans often fixate their attention to a specific scale when looking for objects. Down-sampling can achieve a similar effect by setting the scale for object detection. It effectively creates a pyramid of scales for each image from the previous step.

***Saliency calculation***. Each image from the previous step is now processed by the saliency algorithm. The resulting saliency map can be thought of as an approximate representation of human pre-attentive visual response in a bitmap format. We use Spectral Residual Saliency (RS) approach (Hou and Zhang, 2007) due to its speed and efficiency. The RS method exploits the inverse power law of natural images with the observation that the average of log-spectrums is locally smooth. This enables detecting salient objects based on the log-spectrum of individual images rather than *ensemble* of images. The key steps of this algorithm include:

- Convert the image into the Fourier domain;
- Calculate its phase and amplitude;
- Apply a high pass filter to the log amplitude;
- Transform back into the spatial domain using the modified amplitude and original phase.

These processing steps highlight the modes in the frequency domain, the idea being that objects are defined by boundaries constructed from ridges in the Fourier domain. The output is a set of saliency maps that are further processed to produce individual blobs bracketing objects.

***Region detection***. This step converts each saliency map into detections represented by their attributes (e.g., position, size, orientation, and score). A score is associated with each blob that indicates the confidence score based on its peak saliency value.

***Post-processing and fusion***. This final step uses various parameters, such as, object size, aspect ratio, and score to filter out false positives. In more general situation where there are multiple saliency maps or saliency maps of multiple scales, detections from these multiple maps are fused together with the results from motion to produce a final set of object detections, which will be discussed in Section Detection Fusion.

#### Moving object detection

***Stationary platform***. This module is used to detect foreground (moving) objects from a stationary platform, e.g., tower-mounted camera. The implementation in this work adopts our previous motion-based saliency technique that detects motion by modeling the background and comparing it to the input image (Kim et al., 2005). Key steps in our implementation are described below:

*Codebook generation*. The background model is constructed and updated by adopting our previous work on a codebook-based method (Kim et al., 2005). A codebook consists of one or more codewords that are formed by using samples from each pixel and clustering them based on a color distortion metric and a brightness ratio. A codeword typically contains an RGB-vector and other features such as the minimum and maximum brightness, occurrence frequency, the maximum negative run-length (MNRL) (Kim et al., 2005), and the first and last access times. These values are effectively used to evaluate feature differences, filter out non-background pixels, and recycle unused codewords.

*Color model and filtering*. The color model separates the color and brightness distortion evaluations to handle the problem of illumination changes, for example, shading and highlights. The color and brightness conditions are satisfied only when both the pure colors and brightness lie within acceptable bounds of the codeword. During training, a fat codebook encodes every incoming pixel in the training set. This fat codebook is then filtered to remove codewords that might contain moving foreground objects. We define MNRL as the maximum interval in time that the codeword has not recurred during the training period (Kim et al., 2005). A codeword with a large MNRL is eliminated from the codebook.

*Foreground detection*. To detect foreground in an incoming image frame, each sample pixel is matched against the corresponding background model. Unlike other Gaussian or kernel based methods, this step does not require probability calculations. The codebook method is fast as it only calculates the distance between the sample and the nearest cluster's mean in the codebook. The pixel is classified as foreground if no matching codeword is found; otherwise it is a background. This is followed by region extraction similar to that described in Section Static object detection.

**Figure 4:**
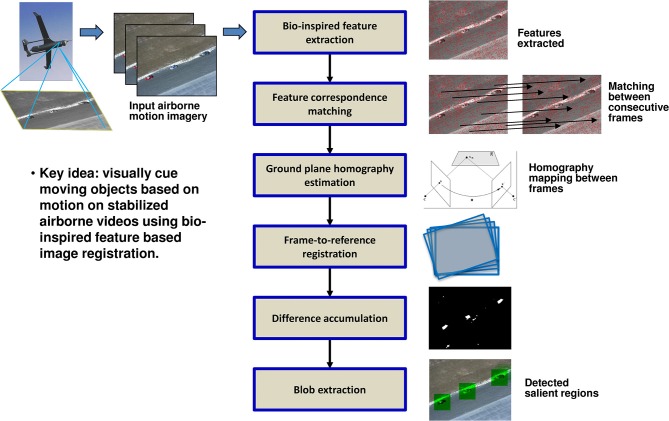
**Block diagram of moving object detection algorithm for airborne motion imagery**.

***Moving platform***. When the sensor platform is moving, we leverage motion information in the video for object detection (Chen et al., 2011; Khosla et al., 2013a). Our approach illustrated in Figure 4 works well even in the presence of jitter and vibration.

*Feature extraction and matching*. Our approach uses Scale Invariant Feature Transform (SIFT) (Lowe, 1999) features due to its attractive properties of invariance to scale, orientation, and affine distortions. SIFT descriptors represent the gradient orientation histograms and can be used to determine similarity between key points. The matching step matches the key points between a pair of images based on Euclidean distance between their SIFT descriptors and outputs a candidate set of matching points.

*Ground-plane homography estimation*. Some of the candidate matches from the previous step may be incorrect due to noise and inherent limitations of SIFT. To remove these false matches, we apply RANSAC (Fischler and Bolles, 1981) which is an iterative method to estimate parameters of a mathematical model from a set of observed data that contains outliers. We use RANSAC to find a ground-plane homography transform that best fits the candidate matches. This provides accurate matches and transformation (i.e., homography) between a pair of images.

*Frame-to-reference registration*. This step warps the frames into a global coordinate reference. Our approach uses a time window size of N frames, with the middle frame being the reference. Each frame is warped to the reference frame in the window based on the homography transformation. This enables all frames in a time window to be stabilized with respect to the center reference frame.

*Difference accumulation*. This step first computes the difference image between the reference frame and each frame registered to it. The difference image corresponds to independently moving objects against the ground plane. This moving pixel detection process accumulates the differences from several registered frames to increase confidence of detection. For example, with window size *N* = 3, the reference frame *F_i_*, its previous frame *F*_*i* − 1_ and next frame *F*_*i* + 1_ are used to compute the frame differences.

*Region extraction*. The previous step effectively produces a motion saliency map, where higher values indicate more prominent motion due to object motion. We then apply the same region extraction method as described in Section Static Object Detection to obtain a set of candidate detections.

#### Detection fusion

The final step in object detection is to combine the form and motion processing detections into a single detection set prior to object classification. Our approach performs fusion within the static object detection followed by its fusion with motion detection. Detections are all represented simply by the detection boxes in terms of their location, size, and score.

For fusion of detections from different saliency channels (e.g., intensity and TCE) and scales, we use several fusion stages. The first stage called “modal” fusion is motivated by observing that different saliency channels carry different importance in a particular application. For example, intensity channel is usually more important than any single channel from TCE, unless the application has a specific goal of finding objects of specific color (e.g., finding all red cars on the road). To account for this we shift the “mode” of detection scores (distribution) by adding an offset to the scores of detections from intensity channel such that detections from intensity channel have higher scores in general than detections from other channels, e.g., a dark channel. After that, fusion of the detections is accomplished by the unions of the detections from different channels, which are prioritized by their scores and will be further filtered in a resource-limited system before they reached the classification stage. The second fusion stage deals with overlapping detections. Detections in different scale can overlap or objects can split split-up in higher resolutions maps. In our implementation, we keep the enclosing and overlapping detections from lower-resolution. In case of detections from different color channels, we keep the detection with the higher score.

**Figure 5:**
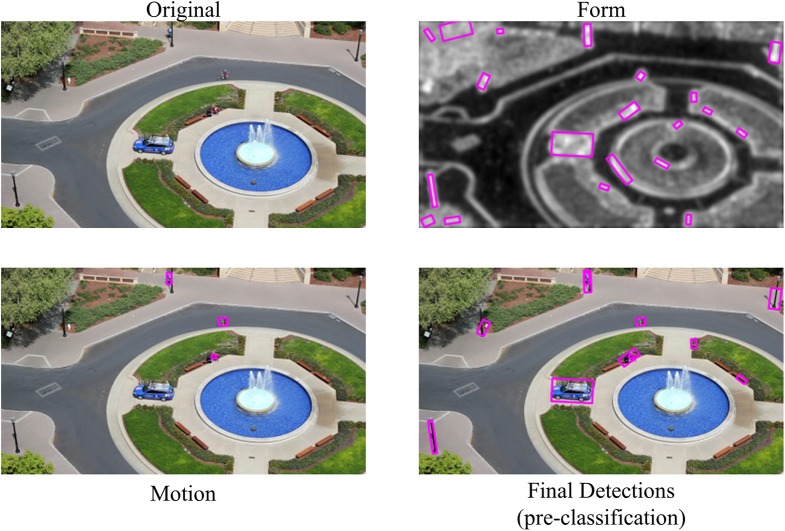
**NEOVUS object detection example by fusion of form and motion pathways**. Input image **(top left)** is processed by separate and parallel pathways for form **(top right)** and motion **(bottom left)**. The detections for each pathway are fused **(bottom right)** and sent to the classification module.

The next step is to merge motion detections with the results from fused detections from static object detection as described above. Here we use a variant of the second fusion scheme described above based on detection overlapping. Our experience is that motion detection is much more reliable than saliency based detection, therefore we keep all detections from motion detection. For the fused detections from static object detection, we only keep them if they do not have significant overlap with any detections from motion detection since such overlap indicates they are the same object. A typical example of the detection process from NEOVUS is shown in Figure 5.

### Object classification

Convolutional neural networks (ConvNets) (LeCun et al., 2010) is a supervised deep-learning neural network with multiple layers of similarly structured convolutional feature extraction operations followed by a linear neural network (NN) classifier. ConvNets are an excellent model for image recognition because the structure allows automatic learning of image features as opposed to engineered ones used in traditional computer vision approaches (Felzenszwalb et al., 2010; Kembhavi et al., 2011). ConvNets typically consist of alternating layers of simple and complex cells as found in mammalian visual cortex. Simple cells perform template matching and complex cells pool these results to achieve invariance. A typical ConvNet has several of 3-layer convolution stages followed by a classifier stage which is a linear NN with one or more hidden layers. Each convolution stage has three layers: (1) a filter bank layer (convolutions) to simulate simple cells (2) non-linearity activation layer, and (3) feature pooling layer to simulate complex cells. The entire network can be trained using backpropagation with stochastic gradient descent (LeCun et al., 2010). Due to its feedforward nature (non-recursive) and uniform computation within each convolution stage, ConvNets are computationally very efficient. It has been implemented in NeuFlow data-flow processor (Farabet et al., 2011) on COTS field programmable gate arrays (FPGAs) to enable low-power, real-time operation. Our prototype hardware evaluation system described in Section Hardware Mapping used the NeuFlow approach.

**Figure 6:**
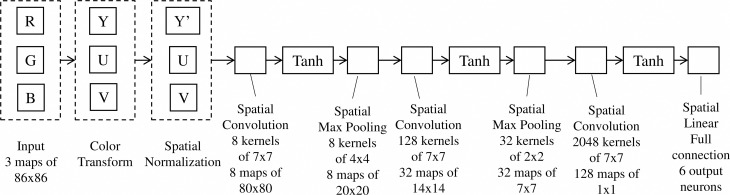
**ConvNet structure implemented in the NEOVUS**.

Our ConvNet implementation follows the traditional architecture outlined above (LeCun et al., 2010) and has three stages. We first resize input RGB color images of candidate target region to 86 by 86 pixels regardless of their original aspect ratio. Then we convert the image to YUV color space, and further process the Y channel by local subtractive and divisive normalization (LeCun et al., 2010). The convolution layer of first stage has eight convolution filter kernels of 7 by 7 pixels, followed by point-wise sigmoid activation function (tanh()) and max-pooling in 4 by 4 pixels neighborhood and subsampling with a stride of 4 pixels, resulting in eight feature maps of 20 by 20 pixels each at the end of stage 1. The remaining stages are detailed in Figure 6. The output of the convolution layer at stage 3 is a 128-D vector, which is then fed to the tanh() non-linear activation layer followed by a fully-connected linear NN classifier. We chose the network parameters based on prior experience with similar datasets and validation.

To train our ConvNet, we use training videos provided by DARPA. Manually annotated video clips were used to train and a separate test set was used to quantify their performance (Section Results and Discussion). These videos contain hand-annotated ground truth for objects of interest in each dataset. The annotated image regions and their class labels are extracted and used to train the ConvNet. Depending on data sets and type of objects, we end up with a few hundreds to a few hundreds of thousands samples for the training step.

## Hardware mapping

**Figure 7:**
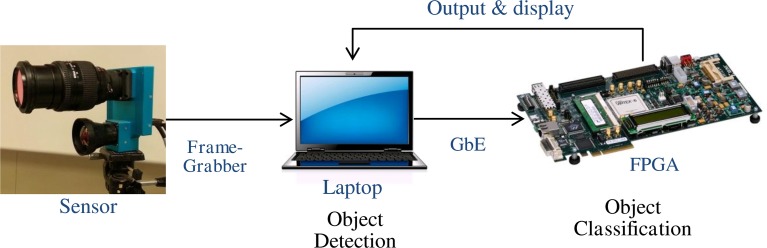
**Block Diagram of the NEOVUS hardware system**. Video frames are captured from the sensor using a frame grabber card and sent to a laptop computer for buffering, display, and initial processing. Classification algorithms are executed on a COTS FPGA board that implements NeuFlow running a pre-trained ConvNet, which accepts image regions and returns a class label to the laptop computer for each region.

The NEOVUS hardware prototype was implemented on commercial COTS hardware (Figure 7) and can process both recorded and live video. For energy evaluation purposes, we processed live video from a 5.6 Megapixel RGB color camera at 30 frames per second. The camera connects to a frame grabber (via CameraLink) and a laptop computer (PCI-Express). The computer runs the object detection algorithm and sends image region corresponding to each detected object to a COTS FPGA board (Xilinx ML605 Virtex-6) for object classification. The FPGA board runs NeuFlow implementation of a trained ConvNet and sends the results to the laptop for subsequent display. The dynamic power of the complete system that includes the 5.6 Mpixel camera, object detection, and object classification components running at 30 frames per second was measured by DARPA during a formal evaluation at 21.7 Watts (W). This translates to effective energy consumption of 5.45 nanoJoules (nJ) per bit of incoming video.

## Results and discussion

**Figure 8:**
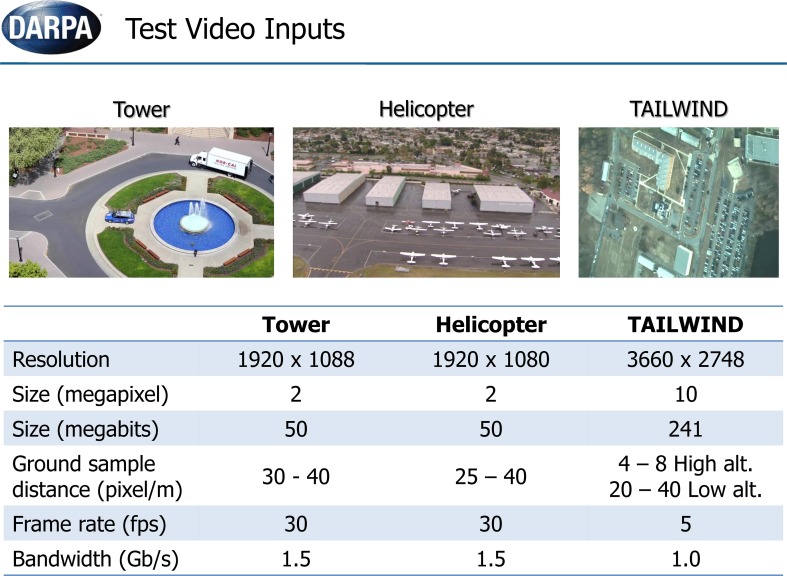
**Three video datasets (Tower, Helicopter, and TAILWIND) were used for the summative tests**.

**Figure 9:**
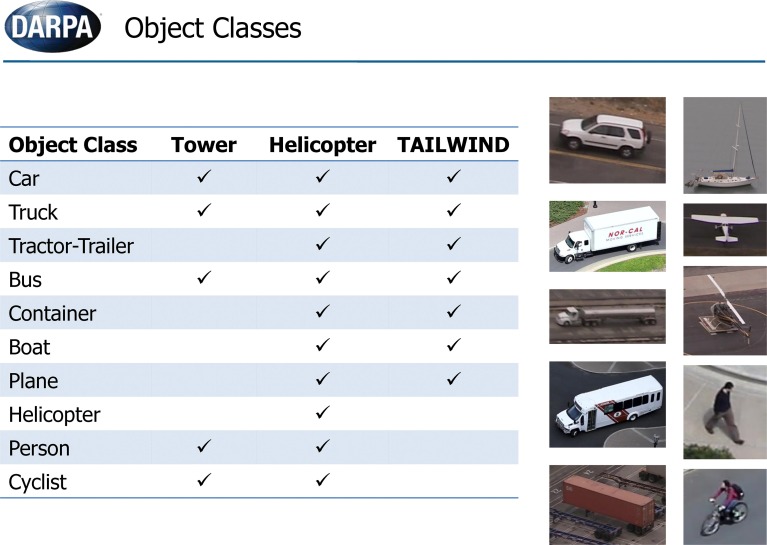
**A maximum of *N* = 10 object classes were to be recognized in the summative tests**.

In this section, we describe results of NEOVUS evaluation by DARPA on three urban area video datasets (Tower, Helicopter and TAILWIND, Figure 8) during summative testing conducted at the end of the Neovision2 program. The Tower dataset is filmed from a fixed camera on top of the Stanford University Hoover tower and the Helicopter dataset is filmed from a helicopter flying over Los Angeles. In both cases, the 1080p video imagery is converted to 8 bit PNG frames for analysis. A third dataset from DARPA TAILWIND (Tactical Aircraft to Increase Long Wave Infrared Nighttime Detection) program is captured from an airplane operating at two different altitudes. The Tower and Helicopter datasets are now available for download (iLab Neovision2 annotated video datasets, 2013a^1^). Each video dataset consists of variable number of object classes (Figure 9). The video frames typically have multiple objects per frame that may be occluded or even overlapping in some cases. The videos are processed through NEOVUS and its outputs in the form of object locations, bounding boxes, and class labels is logged for every frame. The NEOVUS logged results are evaluated using ground-truth and evaluation software. The ground-truth is created by human annotators at VideoMining Corporation who labeled 10 object classes (Boat, Car, Container, Cyclist, Helicopter, Person, Plane, Tractor-Trailer, Bus, and Truck) in these datasets. Each object is enclosed in an oriented rectangle that best encompasses the object. Ten percent of the datasets are annotated by two independent annotators and their outputs are compared to assess quality and consistency of annotations; significant differences between the two sets trigger a review of the annotation process to assure annotation quality. The ground-truth was created under DARPA supervision and no performer in the program had any control of that process. The evaluation software uses Normalized Multiple Object Thresholded Detection Accuracy (NMOTDA) score (Kasturi et al., 2009; iLab Neovision2 Performance Evaluation Protocol, 2013b^2^) to evaluate performance per sequence:

$$NMOTDA=1-\frac{\sum_{i\;=\;1}^{Nframes}\left(c_{m}m^{\left(t\right)}+c_{f}mf^{\left(t\right)}\right)}{\sum_{i\;=\;1}^{Nframes}\left(N_{G}^{\left(t\right)}\right)}$$

where *m^(t)^* = number of missed detections, *f^(t)^* = number of false positives, and *N^(t)^_G_* = number of ground-truth objects in the *t*th frame. The summations are carried out over all evaluated frames. In Neovision2 evaluations, the cost functions *c_m_* and *c_f_* for missed detects and false positives, respectively were set to identity. This is a sequence-based measure which penalizes false detections, missed detections and object fragmentation. Note that maximizing NMOTDA for the sequence is the same as finding the optimal assignment of ground truth boxes to system output boxes at each frame image.

**Figure 10:**
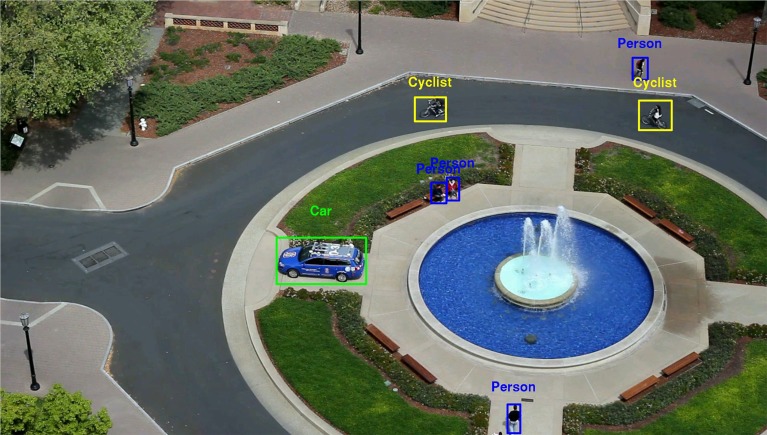
**Sample NEOVUS object classification results on one frame of Tower video dataset (object set: car, cyclist, person, bus, truck)**.

**Figure 11:**
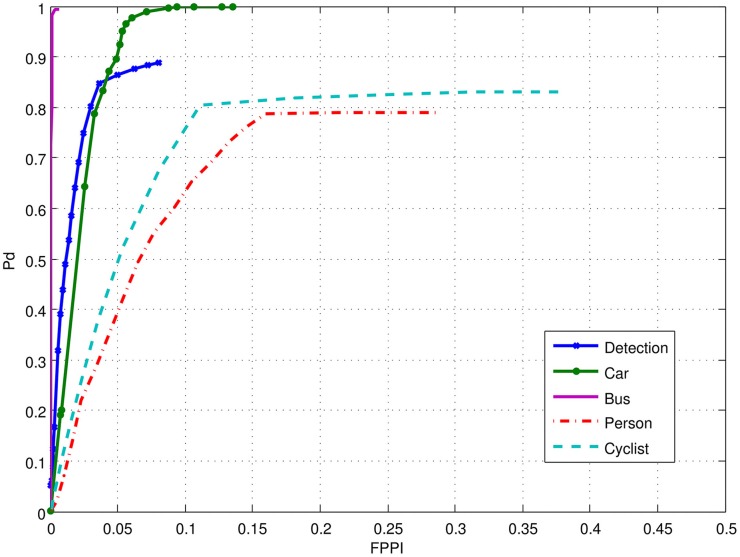
**Performance of the NEOVUS shown as ROCs in terms of P_d_ (probability of correct recognition) vs. False Positives Per Image (FPPI)**. The blue curve “Detection” corresponds to the overall detection performance of all objects regardless of class.

Figure 10 gives an example of NEOVUS results superimposed on a frame of Tower dataset. By aggregating the results, one can plot the ROC curves as P_d_ (probability of correct recognition) vs. FPPI (False Positives Per Image). Figure 11 shows the NEOVUS results for four object classes on the Tower dataset. Overall, NEOVUS achieved excellent object recognition at about 90% per-frame accuracy and 0.1 FPPI.

In each data domain, multiple sequences are used for evaluation. The summary of the performance over the entire dataset, i.e., over all the video clips, is calculated using Weighted NMOTDA (WNMOTDA). WNMOTDA is calculated for each class separately and is given by,

$$WNMOTDA_{i}=\frac{\sum_{j\;=\;1}^{NVC}MOTDA_{ij}*NGT_{ij}}{\sum_{j\;=\;1}^{NVC}NGT_{ij}}$$

where *WNMOTDA_i_* is the NMOTDA for class *i* calculated over all the video clips, *NMOTDA_ij_* is the NMOTDA measure calculated for class *i* in video clip *j, NVC* is the total number of video clips, and *NGT_ij_* is the number of ground truth objects of class *i* in video clip *j*. Finally an average WNMOTDA score is generated for all object classes for each domain by ignoring the class labels. Before scoring, identical boxes are merged into one. Overlapped boxes (if Overlap_Ratio is over 20%) are merged into one and their union is used instead. All of these above calculations are part of the evaluation software.

**Figure 12:**
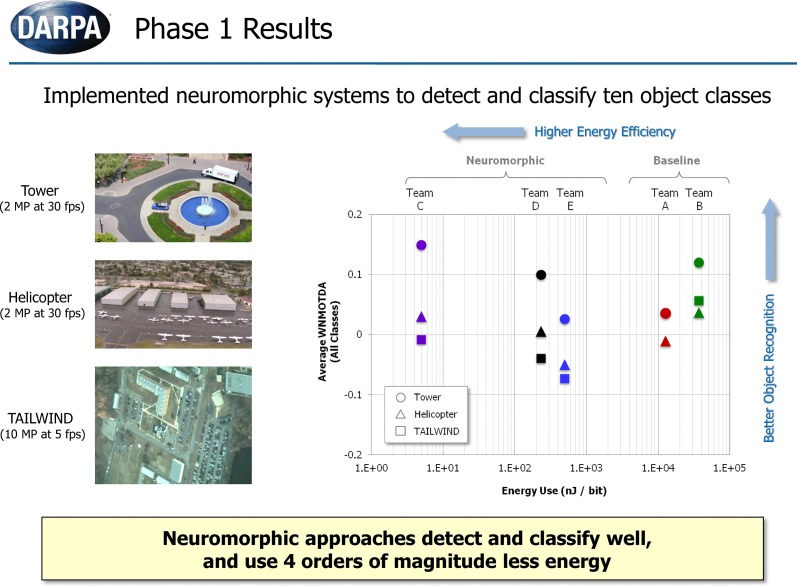
**Results of summative testing released by DARPA**. Y-axis is object recognition performance expressed as WNMOTDA (higher is better). X-axis is energy use expressed as nJ/bit (lower is better). Teams A,B are baseline computer vision teams and teams C–E are neuromorphic teams. HRL's NEOVUS (Team C) was the best performer with high recognition accuracy and at least three orders of magnitude lower energy consumption than state of the art computer vision systems (A,B). (DARPA, 2011)

The recognition performance and energy consumption results of summative testing were formally released by DARPA and are presented in Figure 12 (DARPA, 2011). Five teams participated in the program and evaluation. Three neuromorphic teams (C, D, E) developed neuromorphic vision algorithms. Two baseline algorithms, based on the Deformable Part Model algorithm (Felzenszwalb et al., 2010), representing typical computer vision methods were implemented to serve as a benchmark for comparing against the neuromorphic algorithms. HRL's NEOVUS (Team C) was the best performer with high recognition accuracy (WNOMTDA) and the lowest energy use (nJ/bit) amongst all five teams. Particularly noteworthy is that the NEOVUS energy use was at least three orders of magnitude lower than the computer vision systems (Teams A and B, Figure 12). These unprecedented results show that neuromorphic algorithms and hardware have the potential to revolutionize low-power situational awareness applications, e.g., on-board small unmanned aerial vehicles (UAV).

**Figure 13:**
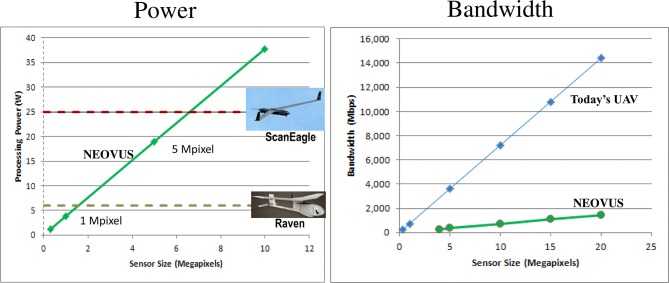
**Projected power consumption and bandwidth of the NEOVUS hardware system**. The power consumption of the NEOVUS hardware system is low enough to even fit onto small UAVs. The bandwidth requirement of the system is far lower than that of a standard UAV surveillance system.

Our current low-power NEOVUS implementation is mature enough to be deployed on mobile platforms toward onboard processing. Figure 13 supports this claim by analyzing platform payload available SWaP and extrapolating our measured energy use (5.45 nanoJoules per bit) to other camera resolutions. For example, NEOVUS could run onboard small UAV's, such as the AeroVironment Raven (Raven RQ-11, 2014^3^), in real-time for a 1-Megapixel camera. Larger UAVs, such as the Boeing-Insitu ScanEagle (Boeing Insitu ScanEagle, 2014^4^), can hold up to a 6-Megapixel camera and still support NEOVUS processing in real-time.

## Conclusions

This work described a neuromorphic object recognition system inspired by neuroscience findings of object recognition pathways in the mammalian visual system. From a practical perspective, the NEOVUS is a proven collection of neuromorphic algorithms, software, and architecture for automated and accurate video object recognition at significantly lower power than state of the art computer vision. It processes video based on human-like search and classification models that are significantly different from computer vision brute-force raster-scan approaches. The NEOVUS was successfully evaluated on real-world urban video datasets and proven to accurately recognize objects at low-power. The successful hardware design and mapping of NEOVUS to COTS hardware can help enable potential autonomous and mobile applications. This onboard processing can reduce both the requirements for data bandwidth and analyst man power. While the NEOVUS hardware was geared for autonomous on-board processing, it is just as applicable to off-board processing of live or recorded images and videos. For off-board processing, the highly efficient algorithms used in NEOVUS can enable video analysis in faster than real-time even with COTS computer hardware.

The NEOVUS described in this work is a feed-forward, bottom-up object recognition architecture. However, models and algorithms for top-down attention and processing can be added to the current architecture with little modifications. For example, goal-directed search and classification, e.g., find and track all white trucks) can be added to our framework. Future work will include these top-down aspects and onboard evaluation of this capability. This is expected to yield the greatest level of improvement toward enabling practical systems.

### Conflict of interest statement

The authors declare that the research was conducted in the absence of any commercial or financial relationships that could be construed as a potential conflict of interest.

## Footnotes

^1^Available online at: http://ilab.usc.edu/neo2/dataset/

^2^Available online at: http://ilab.usc.edu/neo2/dataset/neovision2-performance-evaluation-protocol.pdf

^3^Available online at: http://www.avinc.com/uas/adc/raven/.

^4^Available online at: http://www.insitu.com/systems/scaneagle
